# Combined organic biomarker and use-wear analyses of stone artefacts from Liang Bua, Flores, Indonesia

**DOI:** 10.1038/s41598-019-53782-2

**Published:** 2019-11-26

**Authors:** Susan Luong, Matthew W. Tocheri, Elspeth Hayes, Thomas Sutikna, Richard Fullagar, E. Wahyu Saptomo, Jatmiko, Richard G. Roberts

**Affiliations:** 1 Centre for Archaeological Science, School of Earth, Atmospheric and Life Sciences, University of Wollongong, Wollongong, New South Wales 2522 Australia; 2 Department of Anthropology, Lakehead University, Thunder Bay, Ontario P7B 5E1 Canada; 3 Human Origins Program, Department of Anthropology, National Museum of Natural History, Smithsonian Institution, Washington, DC 20013 USA; 4 Australian Research Council Centre of Excellence for Australian Biodiversity and Heritage, University of Wollongong, Wollongong, New South Wales 2522 Australia; 5 Pusat Penelitian Arkeologi Nasional, Jakarta, 12510 Indonesia

**Keywords:** Analytical chemistry, Environmental sciences

## Abstract

Organic biomarker and lithic use-wear analyses of archaeological implements manufactured and/or used by hominins in the past offers a means of assessing how prehistoric peoples utilised natural resources. Currently, most studies focus on one of these techniques, rather than using both in sequence. This study aims to assess the potential of combining both methods to analyse stone artefacts, using a set of 69 stones excavated from the cave site of Liang Bua (Flores, Indonesia). Prior to chemical analysis, an initial inspection of the artefacts revealed potential use-wear traces but no visible residues. Gas chromatography mass spectrometry (GC-MS) analysis, including the targeting of 86 lipids, terpenes, terpenoids, alkanes and their analogues, found compounds with plant or animal origin on 27 of the 69 stones. The artefacts were subsequently cleaned, and use-wear analysis identified traces of use on 43 artefacts. Use-wear analysis confirmed traces of use on 23 of the 27 artefacts with potential use-residues that were determined by GC-MS. The GC-MS results were broadly consistent with the functional classes identified in the later use-wear analysis. This inclusive approach for stone artefact analysis strengthens the identifications made through multiple lines of enquiry. There remain conflicts and uncertainties in specific cases, suggesting the need for further refinement and analyses of the relationships between use-wear and residues.

## Introduction

Evidence of the former function(s) of archaeological artefacts includes visible use-wear traces and/or preserved residues, which may be incorporated within the fabric of the artefact (e.g., pottery) or adhered to one of the artefact’s surfaces (e.g., stone tools). To determine the origins of potentially ancient residues, organic compounds such as lipids, terpenes, terpenoids, alkanes, proteins and carbohydrates are often the analytes of interest in chromatographic/mass spectrometric analyses^1–8^. If the residue can be reliably and accurately related to past use, then the function(s) of the implements can be logically deduced, providing insights into the behaviours of the tool users. To increase confidence in the characterisation of residues and to aid in their source identification, organic biomarkers can be targeted from various compound classes to obtain information on the types of analytes present and their combination (i.e., a chemical profile). Separating residues with archaeological significance from environmental contamination is not straightforward, however, owing to the often-complex depositional context of archaeological artefacts. Consequently, in order to make reliable inferences it is necessary to analyse a relatively large sample set of artefacts and to monitor for different groups of analytes that are expected to be present in the original materials applied to the artefacts.

Improved understanding of the chemical processes operating in the proximity of potential use-residues is also important to narrow down the list of possible sources of residues. The extent of residue degradation and the molecular transformation pathways followed is significantly influenced by the depositional environment. In general, residue preservation is more likely in dry rather than in moist environments^1,5,9,10^. In the case of lipids, short chain fatty acids^9^ and fatty acid oxidation products (such as dicarboxylic acids, hydroxy acids and keto acids) are prone to removal from artefacts by percolating groundwater^11^. The presence of the acidic carboxylic acid functional group makes these acids capable of carrying a charge, rendering them relatively hydrophilic and more mobile in water compared to neutral lipids, such as triacylglycerols and wax esters. Wet environments may aid hydrolysis^1^, however, resulting in cleavage of the original molecule. Degradation studies have shown that anoxic burial conditions are more favourable than oxic environments for lipid preservation^12^. Microbial growth is suppressed in desiccated environments, but abiotic oxidative processes can still occur^4^. Other environmental parameters that affect preservation include the degree of light exposure, temperature and redox conditions^4^.

In this study, we used a sensitive and selective analytical workflow involving gas chromatography tandem mass spectrometry (GC-MS/MS) to conduct multiresidue analysis of solvent extracts from 69 stones (including 68 artefacts and one non-artefact) excavated from Liang Bua, a cave site on the Indonesian island of Flores^13–17^. The deposits contain the skeletal remains of anatomically modern humans (*Homo sapiens*) and another hominin species, *Homo floresiensis*, as well as artefacts attributed to both of these taxa. The artefacts studied here are associated only with anatomically modern humans, and originate from deposits dated to between about 19 and 1 thousand years ago (ka).

Chemical profiles of the residues were constructed by monitoring non-volatile and volatile low molecular weight organic compounds (<1000 Da) in the extracts (including 86 lipids, terpenes, terpenoids, alkanes and their analogues) using two validated tandem (targeted) GC-MS methods and two non-targeted GC-MS methods to satisfy the requirements for robust residue MS analysis of artefacts^7,8^. In ‘non-targeted’ mode, the instrument looks for all compounds within a given scanning mass range set by the analyst, not just for specific compounds (as with ‘targeted’ analysis). These chemical profiles were constructed and applied to discriminate between unused and potentially-used artefacts, and group the latter residues according to their most probable origins (plant, animal or both), based on the presence or absence of sterols, terpenoids and terpenes (the more taxonomically-specific analytes in the suite of biomarkers monitored). The collection of non-targeted GC-MS data for the solvent extracts enabled retrospective analyses of any compounds that were not incorporated into the targeted methods.

The primary purpose of the current study is to apply the most appropriate advanced analytical techniques, combined with lithic use-wear analysis, to investigate the chemistry of organic residues on stone artefacts in a tropical environmental setting, and to evaluate agreement/disagreement between the chemistry and use-wear results. Although essentially a proof-of-concept study, the combined MS and MS/MS data were used to draw preliminary inferences about the resource processing practices of prehistoric modern human populations at Liang Bua and the depositional context of these stone artefacts. This analytical workflow is potentially applicable to archaeological implements excavated from other sites worldwide and can be used to analyse other types of artefacts.

## Methodology

The GC-MS/MS portion of our methodology has been published previously. A comprehensive analytical workflow was developed to monitor and quantify non-volatile low molecular weight lipids on 14 stone artefacts from Liang Bua using GC–MS/MS^7^. In a subsequent publication^8^, a GC–MS/MS method for the detection and quantification of terpenes, monoterpenoids and alkanes was developed, validated (in line with acceptable analytical practices^18^) and applied to several stone artefacts from the 2017 study. GC-MS/MS was applied here as it is a more sensitive and selective technique compared to conventional GC-MS. This allowed us to detect compounds at significantly lower quantities and to differentiate between very similar compounds with greater confidence.

Conventional use-wear analysis requires artefact cleaning and handling that is inimical to reliable GC-MS detection of significant archaeological residues, so GC-MS analysis must be performed first. However, before artefact cleaning, sufficient sediment is often removed during excavation to enable observation of some edges and the presence/absence of developed use-wear (e.g., edge-rounding, edge-gloss and retouch) either macroscopically or under low magnification. Nevertheless, subsequent use-wear analysis is essential to confirm mode of use and what materials were processed. Consequently, this methodology allows for discussion here that compares three data sets: (1) presence/absence of use-wear identified during initial inspection, before cleaning, (2) chemical profiles of used and unused artefacts, and (3) use-wear analysis after cleaning.

### Archaeological context, stone artefact selection and initial inspection

Previous analyses suggest that the stone artefacts made and used by *H. floresiensis* ~190–50 ka^16,17^ are broadly similar to those from Oldowan/Developed Oldowan assemblages in eastern Africa in both morphology and technology^19^. These artefacts are also essentially similar to those made and used by anatomically modern humans at Liang Bua from their earliest arrival until ~3–4 ka, when rectangular-sectioned stone adzes are first recorded^20^. The primary stone flaking and reduction strategies used at Liang Bua by both of these hominin species involved freehand hard hammer percussion, as well as burination, truncation and bipolar techniques^20^. Moreover, the stone artefact assemblages attributed to *H. floresiensis* are distinguished from those made by anatomically modern humans at the site by only three main characteristics: exposure to fire, raw material selection, and presence of edge-gloss^17,20^. Approximately 18% of the modern human stone artefacts shows evidence of exposure to fire, whereas less than 1% of the *H. floresiensis* assemblage shows the same^20^. Further, the modern human assemblage includes a significantly greater proportion of chert artefacts than in the *H. floresiensis* assemblage, which is instead dominated by artefacts made of silicified tuff^17,20^. Silicified tuff, a type of volcanic rock, is the most abundant raw material at Liang Bua and its surrounding area^17^. This suggests that anatomically modern humans may have selectively targeted chert sources located further away and then transported these materials to the cave, whereas *H. floresiensis* did not and instead used stones readily available from the cave’s vicinity^17^. Finally, the only evidence thus far of edge-glossed flakes occurs among the modern human stone artefacts^20^.

Additional lines of evidence extracted from the Liang Bua stone artefacts would greatly extend comparisons between the behaviours of *H. floresiensis* and anatomically modern humans, as well as possibly elucidating further hominin behavioural changes through time. Thus, our study is focussed on the methodological issue of evaluating a new approach to residue analysis that has the potential for rapid processing of large samples of artefacts that may ultimately provide critical information about specific archaeological questions (e.g., resource use, lithic variability, associated cultural changes). The artefacts targeted for this study, however, were not intended to form a representative sample of stone tools within or between stratigraphic units at Liang Bua, but rather as a manageable selection that was collected under appropriate conditions and enables an assessment of the viability of the combined GC-MS/use-wear methodology employed here.

**Figure 1:**
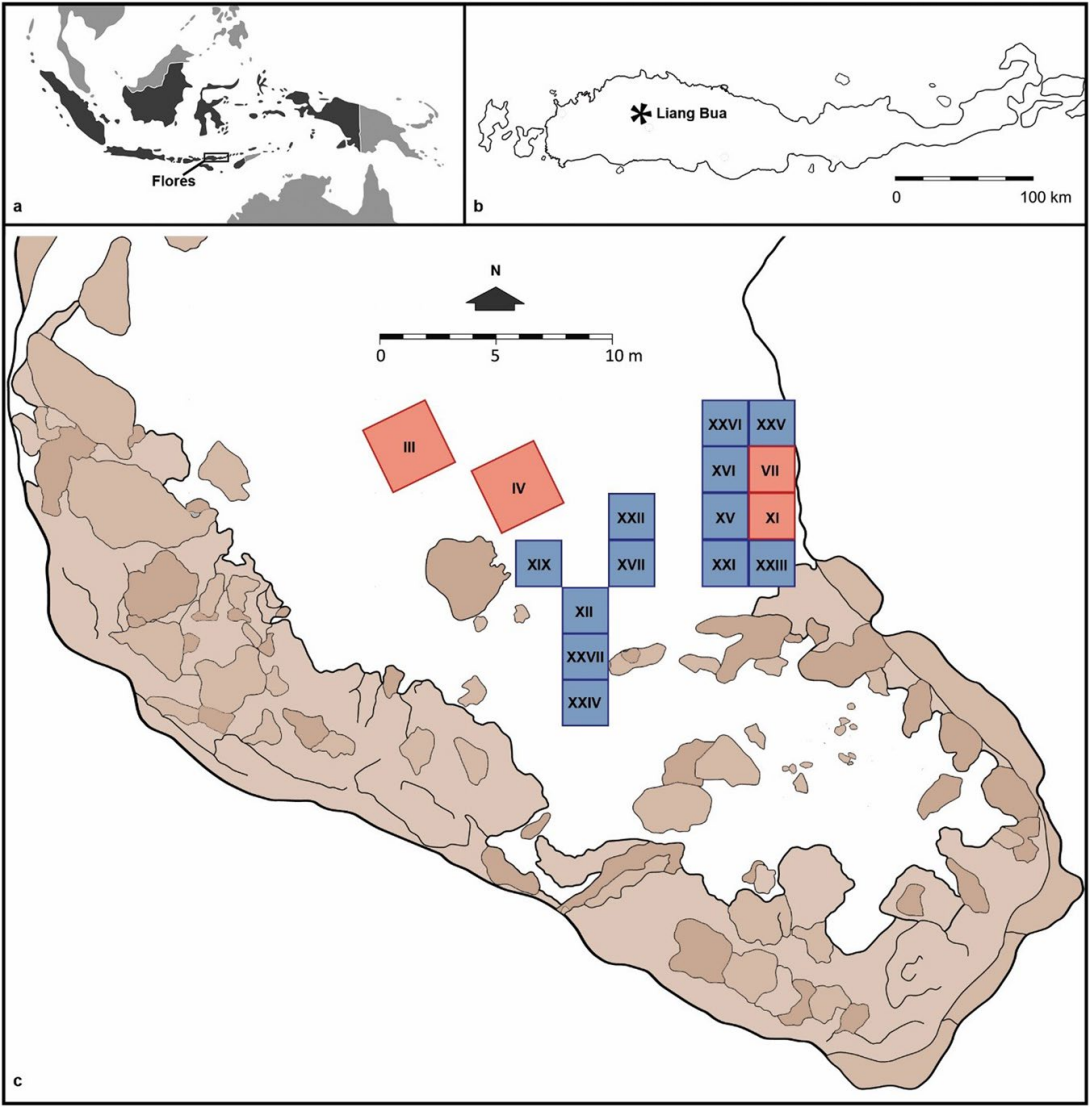
Site location: (**a**) location of Flores within Indonesia, (**b**) location of Liang Bua on Flores, and (**c**) site plan of main areas excavated previously; Roman numerals denote the Sector numbers designated by the National Research Centre for Archaeology in Indonesia (modified from Sutikna *et al*., 2016). The 2001–2004 and 2007–2015 excavations are shaded red and blue, respectively, while the remaining cave floor sediments are shaded white, the areas shaded brown are exposed rocks, stalagmites and other surfaces covered in speleothems. The stone artefacts that are the focus of the present study were excavated in 2015 from Sectors XXIV, XXV and XXVI.

The stone artefacts analysed in this study were excavated in 2015 from Sectors XXIV, XXV and XXVI (Fig. 1 and Table 1). The stones derive from stratigraphic units 6 and 8, which have been dated to around 18–13 and 12–0 ka, respectively, using 63 recovered charcoal samples^17^. During excavation in the field, these artefacts were minimally exposed using metal trowels, and each was collected on and often firmly encased within a surrounding pedestal of sediment. The whole pedestal was then wrapped in plastic film. All the artefacts on pedestals arrived in the laboratory in an uncleaned state with sediment attached and were subsequently excavated from the pedestals using a small metal spatula. Thus, each artefact was recorded and catalogued under clean laboratory conditions to control for and minimise cross-contamination. For example, the only materials that came into contact with the artefacts during the laboratory excavation, initial inspection, documentation and use-wear inspection were individually sealed plastic storage bags and starch-free nitrile gloves, which were replaced following the handling of each artefact. Each artefact was inspected macroscopically and at low magnification (×6.7 to ×45) using an Olympus SZ61 stereomicroscope with an external fibre optic, 150 Watt halogen light source (Olympus LG-PS2) to provisionally record stone material, artefact class and any visible traces of use.

**Table 1:** Recovery location, extraction group assignment, raw material, technological class, approximate age range and evaluation of use traces for the artefacts selected for this study.

Artefact^a^	Depth^b^ (cm)	Extraction group^c,d^	Raw material	Technological class	Stratigraphic unit	Age range^e^ (ka)	Use-wear traces noted during initial macroscopic/low-magnification inspection, before cleaning	Use-wear analysis under low- and high-magnification, after cleaning
XXIV-43	60	G1	quartz	non-artefact	8	5–1	absent	unused
XXIV-42	62	G1	silicified tuff	flake	8	5–1	absent	definite use, but unsure of the material processed
XXIV-79	63	G1	chalcedony	broken flake	8	11–5	absent	unused^h^
XXIV-56	71	G1	silicified tuff	broken retouched flake	8	5–1	absent	definite use, but unsure of the material processed
XXIV-69	76	G1	chert	flake	8	11–5	absent	definite use, includes plant
XXV-3923	504	G1	chert	flake	6	14.01–11.75	absent	possible use
XXV-3951	516	G2	andesite	flake from pounding stone or anvil (no used flake margin)	6	14.01–11.75	absent	definite use from pounding, but unsure of the material processed (possibly mineral pigment)
XXV-3950	519	G2	chert	core	6	14.01–11.75	absent	unused
XXV-3957	522	G1	silicified tuff	broken flake	6	14.01–11.75	absent	unused
XXV-3959	522	G2	chert	core	6	14.01–11.75	absent	unused
XXV-4022	528	G2	silicified tuff	flake	6	14.01–11.75	absent	probable use
XXV-4128	538	G1	chalcedony	broken flake	6	14.01–11.75	absent	unused
XXV-4130	539	G1	chert	broken flake	6	14.01–11.75	absent	unused
XXV-4132	542	G2	chert	broken flake	6	14.01–11.75	absent	unused
XXV-4203	554	G1	chalcedony	retouched flake	6	14.01–11.75	absent	possible use^h^
XXV-4254	560	G1	silicified tuff	flake	6	14.01–11.75	absent	unused
XXV-4253	562	G1	chalcedony	flake	6	14.01–11.75	absent	unused
XXV-4286	566	G1	silicified tuff	broken flake	6	14.01–11.75	absent	unused
XXV-4283	571	G1	chert	flake (burnt)	6	14.01–11.75	absent	probable use
XXVI-4338	534	G1	chert	flake	6	14.01–11.75	absent	unused
XXVI-4410	538	G2	chalcedony	core	6	14.01–11.75	absent	unused
XXVI-4413	538	G1	chert	flake	6	14.01–11.75	absent	probable use
XXVI-4531	551	G2	silicified tuff	core	6	14.01–11.75	absent	unused^h^
XXVI-4581-A^f^	558	G1	silicified tuff	retouched flake	6	14.01–11.75	absent	possible use
XXVI-4581-B^f^	558	G1	chert	debris	6	14.01–11.75	absent	unused
XXVI-4655	584	G2	andesite	broken hammerstone	6	18.58–13.75	absent	definite use as hammer (stone on stone)
XXVI-4963	623	G2	silicified tuff	flake	6	18.97–17.45	absent	unused
XXVI-4964	625	G1	silicified tuff	flake	6	18.97–17.45	absent	probable use
XXVI-4965	625	G2	silicified tuff	retouched flake	6	18.97–17.45	absent	possible use^h^
XXVI-4966-A^f^	625	G1	chert	broken retouched flake	6	18.97–17.45	absent	definite use, but unsure of the material processed^h^
XXVI-4966-B^f^	625	G1	chert	flake	6	18.97–17.45	absent	unused
XXVI-4828	630	G1	silicified tuff	broken flake	6	18.97–17.45	absent	unused
XXVI-5004-A^f^	630	G1	chert	broken retouched flake	6	18.97–17.45	absent	probable use
XXVI-5004-B^f^	630	G1	silicified tuff	flake	6	18.97–17.45	absent	unused
XXVI-5045	637	G2	silicified tuff	flake	6	18.97–17.45	absent	unused
XXVI-5046	637	G1	chert	broken retouched flake	6	18.97–17.45	absent	definite use, but unsure of the material processed
XXVI-5047	638	G1	chert	flake	6	18.97–17.45	absent	unused
XXVI-5048	638	G1	silicified tuff	broken flake	6	18.97–17.45	absent	unused^h^
XXVI-5106	646	G1	silicified tuff	flake	6	18.97–17.45	absent	possible use
XXVI-5107	649	G1	silicified tuff	flake	6	18.97–17.45	absent	unused
XXVI-5108	649	G2	silicified tuff	flake	6	18.97–17.45	absent	probable use
XXVI-5135	657	G1	silicified tuff	non-diagnostic artefact	6	18.97–17.45	absent	possible use
XXVI-5151	662	G2	chert	flake	6	18.97–17.45	absent	definite use, but unsure of the material processed
XXIV-68	77	G3	chert	broken retouched flake	8	11–5	present	probable use
XXIV-67	79	G3	silicified tuff	flake	8	11–5	present	unused
XXV-3932	509	G3	chert	flake	6	14.01–11.75	present	possible use
XXV-3931	513	G5	chalcedony	flake	6	14.01–11.75	present	definite use, and the material (plant) can be identified by use-wear
XXV-3955	517	G4	silicified tuff	flake	6	14.01–11.75	present	possible use
XXV-3953	521	G5	silicified tuff	retouched flake	6	14.01–11.75	present	possible use
XXV-3956	521	G4	chert	flake	6	14.01–11.75	present	possible use
XXV-3954^g^	523	G4	chert	flake	6	14.01–11.75	present	definite use, and the material (plant) can be identified by use-wear
XXV-4025^g^	526	G5	silicified tuff	broken retouched flake	6	14.01–11.75	present	probable use
XXV-4256	558	G4	silicified tuff	flake	6	14.01–11.75	present	probable use
XXV-4255	561	G4	chert	broken flake	6	14.01–11.75	present	definite use, but unsure of the material processed^i^
XXV-4443	618	G4	chert	broken flake	6	18.58–13.75	present	definite use, but unsure of the material processed^i^
XXV-4619	629	G3	chert	broken flake	6	18.97–17.45	present	probable use
XXVI-4257	525	G3	chert	retouched flake	6	14.01–11.75	present	definite use, and the material (plant) can be identified by use-wear
XXVI-4337	530	G5	jasper	retouched flake	6	14.01–11.75	present	unused^i^
XXVI-4414	539	G4	chert	flake	6	14.01–11.75	present	possible use
XXVI-4411	541	G5	chert	retouched flake	6	14.01–11.75	present	definite use, but unsure of the material processed
XXVI-4412	541	G3	chert	broken flake	6	14.01–11.75	present	definite use, but unsure of the material processed
XXVI-4534-A^f,g^	552	G5	silicified tuff	flake	6	14.01–11.75	present	possible use^i^
XXVI-4534-B^f^	552	G3	quartz	broken flake	6	14.01–11.75	present	unused^i^
XXVI-4532	554	G5	chert	retouched flake	6	14.01–11.75	present	definite use, but unsure of the material processed
XXVI-4533-A^f^	554	G3	jasper	core	6	14.01–11.75	present	possible use
XXVI-4533-B^f^	554	G3	silicified tuff	broken flake	6	14.01–11.75	present	definite use, but unsure of the material processed
XXVI-4579	557	G3	chert	flake	6	14.01–11.75	present	possible use
XXVI-4583	560	G3	chalcedony	broken flake	6	14.01–11.75	present	possible use
XXVI-5002^g^	627	G4	silicified tuff	flake	6	18.97–17.45	present	definite use, but unsure of the material processed

^a^The excavated Sector number is given in Roman numerals, and the subsequent number refers to the sequential order in which the artefact was excavated/recorded within each respective Sector.

^b^The depth each artefact was recovered as measured from the present cave floor surface for each respective Sector.

^c^For artefacts where traces of use are absent after initial inspection, G1 (n = 30) and G2 (n = 13) specimens required 16–30 mL and 50–100 mL of extraction solvent for total submersion, respectively.

^d^For artefacts where traces of use are present after initial inspection, the volume ranges used for edge-of-interest (E1) and total submersion (E2) extractions were: 1–4 mL and 4–40 mL, respectively, for G3 specimens; 4–8 mL and 30–70 mL, respectively, for G4 specimens; and 4–30 mL and 50–120 mL, respectively, for G5 specimens.

^e^For Sectors XXV and XXVI, approximate age ranges are based on calibrated radiocarbon ages (in thousands of years before AD 1950) obtained from charcoal recovered *in situ* during excavation of sediments stratigraphically above and below the stone artefacts (upper and lower 95% confidence intervals for the oldest and youngest ages, respectively)^17^. For Sector XXIV, approximate age ranges are based on biostratigraphy and calibrated radiocarbon ages reported in Morley *et al*.^35^.

^f^On occasion, two stone artefacts were found in the same sediment pedestal; ‘A’ refers to the first artefact that was partially exposed during excavation, and ‘B’ refers to the second artefact that was initially concealed within the pedestal and only revealed within the laboratory.

^g^Specimen with more than one edge with traces of use.

^h^Artefacts with no provisional traces of use, containing either β-sitosterol, abietic acid or cholesterol detectable by GC-MS.

^i^Artefacts with provisional traces of use, yielding no taxonomically-specific analytes that were monitored by GC-MS.

During this initial inspection and recording (including photography before and after removal), each specimen was classified as either a flake, core, pounding stone or non-artefact, and wear traces were noted as absent (n = 43) or present (n = 26) (Table 1).

These initial observations of potential traces of use are not a substitute for use-wear analysis (which was instead undertaken after the GC-MS residue analysis), but are included here because they raise interesting issues about interpretations of residues and tool function. After initial inspection, the artefacts were then immediately subjected to GC-MS analysis in order to minimise contamination and removal of potentially archaeologically significant residues. Subsequent to GC-MS analysis, the artefacts were cleaned with distilled water and then with ethanol, and subjected to use-wear analysis under both low- and high-magnification.

### GC-MS organic biomarker analysis of solvent extracts

**Figure 2:**
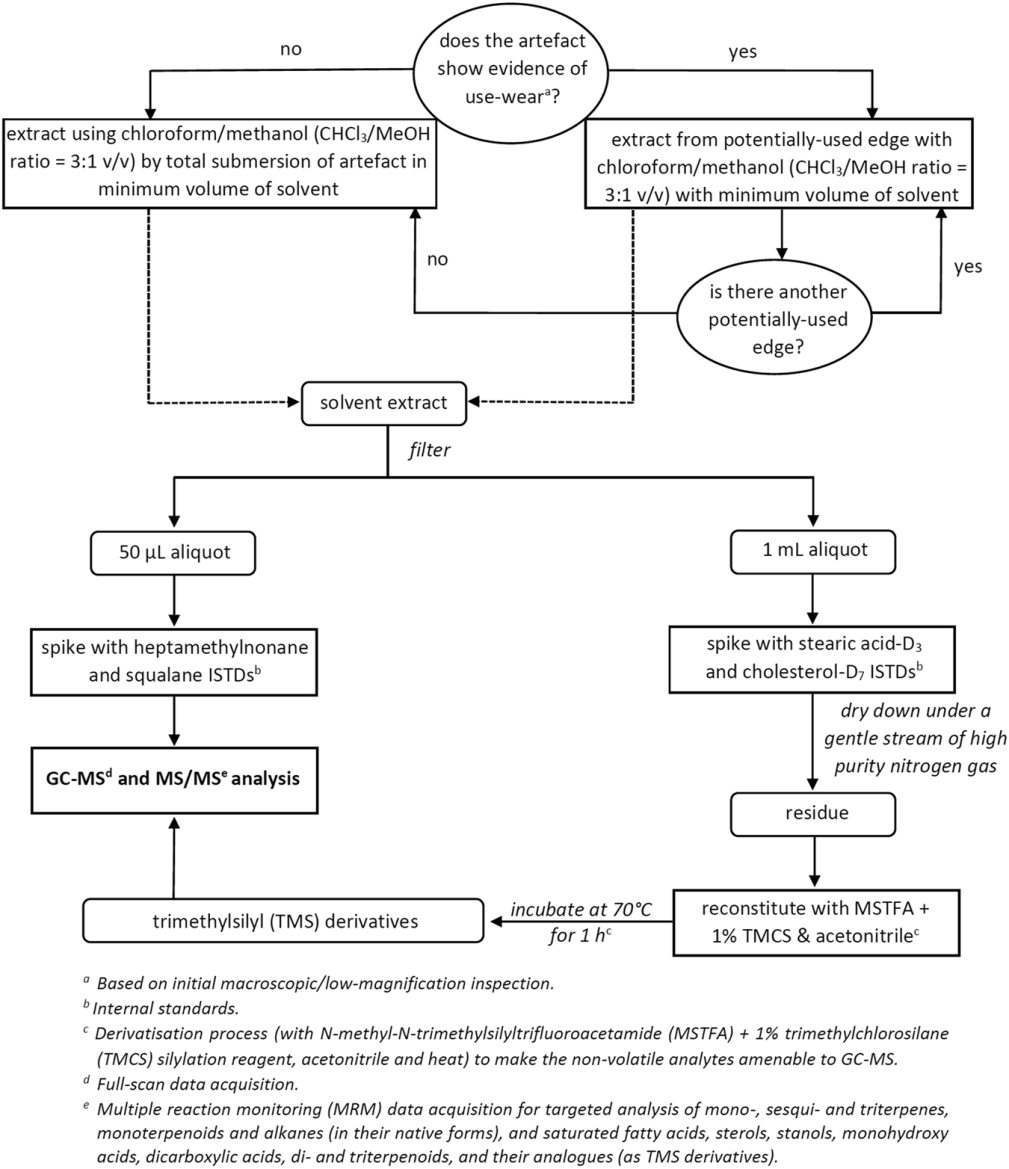
Flowchart outlining the analytical workflow used for low molecular weight organic biomarker analysis of stone artefacts from Liang Bua.

Solvent extracts from the stone artefacts were analysed for non-volatile low molecular weight lipids and volatile terpenes, monoterpenoids and alkanes according to an analytical workflow specifically developed for this task. This included a fit-for-purpose solvent extraction technique, two targeted GC-MS/MS methods and two non-targeted GC-MS methods (Fig. 2). The first targeted (multiple reaction monitoring, MRM) method quantified non-volatile low molecular weight lipids, as their trimethylsilyl derivatives; a non-targeted (full-scan) method was also used to monitor the samples for other analytes amenable to GC-MS by the derivatisation process, but were not included in the targeted method. The second targeted (MRM) method detected and quantified terpenes, monoterpenoids and alkanes, as the native species, and a corresponding non-targeted (full-scan) method was used to scan the samples for similar analytes. The targeted analytes could not be combined into one targeted method, as the derivatisation process degraded the terpene/terpenoid compounds. Target compound monitoring increased sensitivity for the quantitative analysis. However, the full-scan acquisition portion of the analyses allowed us to look at the data retrospectively, to search for compounds that may be of interest later (e.g., after acquiring new knowledge relating to stone tool function, from use-wear or spectroscopic analyses), but was not incorporated into the targeted methods at the time of analysis.

Residues were removed from the stone artefact surfaces using a 20 min solvent extraction with chloroform/methanol (3:1 v/v), assisted by ultrasonication. Artefacts that showed no provisional traces of use were covered in the minimum volume of extraction solvent required for total submersion. Artefacts that showed provisional traces of use underwent a double or triple extraction. For each artefact, the edge(s) with provisional traces of use were first extracted. The artefact was then totally submerged in the minimum volume of solvent, in the same manner as with the artefacts with no provisional traces of use. The protocol for analysing extracts from partial and total immersions was identical, and comparison of partial and total immersion results are valid, since, from a chemical perspective, the prerequisite for potential use was the presence and positive identification of particular compounds on each artefact (the concentrations do not matter at this stage). Further, the various partial and total immersions of artefacts with no traces of use and non-artefacts provided ‘controls’ to determine chemical profiles for stones and surrounding sediment (i.e., environmental controls).

The extracts were filtered through a 0.22 µm hydrophobic syringe filter unit (MicroAnalytix: Taren Point, NSW, Australia) and processed for GC-MS analysis. The solvent volume ranges required for the extractions vary because of artefact size, and artefacts were grouped into five extraction groups (G1–G5), as shown in Table 1. All residue extracts, including those from each total immersion and each partial immersion of artefacts, were subjected to identical sample work-up protocols as shown in the analytical workflow (Fig. 2). In addition to the artefact extracts, calibration sets each consisting of a reagent blank, calibrator and quality control (QC) standards were also prepared and analysed. An Agilent Technologies 7890 GC system coupled to a 7000 GC/MS triple quadrupole mass spectrometer was used for the analysis of all artefact extracts, calibrator standards, QC standards and reagent control samples. Chromatographic separation was carried out using an Rxi-5Sil MS 1,4-bis(dimethylsiloxy)phenylene dimethyl polysiloxane fused silica column (20 m × 0.18 mm × 0.18 µm; Restek: Bellefonte, Pennsylvania). Please refer to Luong *et al*.^7,8^ for full details relating to the instrumental parameters (for both full-scan and MRM data acquisition) and analyte identification, quantification and data processing procedures using the Agilent MassHunter qualitative and quantitative software packages.

### Use-wear analysis

After completion of the chemical residue analysis, use-wear analysis identified the location and extent of the main types of use-wear, including scarring or edge fracturing, striation, polish and smoothing, and edge-rounding (after Kamminga^21^), following standard procedures (e.g., Fullagar^22^). Artefacts were observed under low magnification (×6.7 to ×45) using (1) an Olympus SZ61 stereomicroscope with an external fibre optic, 150 Watt halogen light source (Olympus LG-PS2) with an Olympus Infinity 2 digital camera; and (2) a Leica MZ16A stereomicroscope with an automatic Z-stacking function from multifocal images obtained using a DFC320 Leica camera and Leica LAS V4.4 software. Artefacts were also examined using an Olympus metallographic microscope (model BX51) with vertical incident light (with brightfield/darkfield lighting and polarising filters) and long working distance objective lenses (×50, ×100, ×200 and ×500); images were captured with an Olympus Infinity 2 camera.

The analysts’ level of confidence in interpreting the function of each artefact is indicated by four terms in the use-wear summary (Table 1). ‘Unused’ refers to high confidence that the artefact has not been used. ‘Definite use’ indicates high confidence that that artefact has been used, usually on a particular class of material (e.g., scraping siliceous plant or bone). ‘Possible use’ and ‘Probable use’ refer to lower and higher levels, respectively, of confidence that an artefact has been used, but indicate a degree of uncertainty because of (a) a relatively low development of polish and other forms of use-wear, and (b) the possible overlap with patterns of surface wear, edge damage and other alterations caused by weathering and other post-depositional processes.

## Results and Discussion

### Initial observations prior to cleaning and use-wear analysis after cleaning

As expected, initial artefact inspection greatly underestimated the number of artefacts with traces of use identified after cleaning and use-wear analysis. Prior to cleaning, initial low-magnification microscopy showed that traces of use were present on 26 artefacts and absent on 43 artefacts. After cleaning and use-wear analysis (stereozoom and metallographic microscopy), traces of use were identified on 43 artefacts, (including 18 with high confidence of use) and no traces of use were identified on 26 artefacts (Tables 1 and S1). However, the discrepancies raise some interesting and unresolved issues, particularly about the interpretation of use-wear and residues.

Of the 26 artefacts with traces of use noted in the initial inspection, three (XXIV-67, XXVI-4337 and XXVI-4534-B) were interpreted as definitely unused after the use-wear analysis (i.e., high confidence that the tools were not used) (Table 1). The interpretations of two of these artefacts as unused (XXVI-4337, a flake; and XXVI-4534-B, a small broken flake) were supported by the absence of the relatively taxonomically-specific analytes (i.e., sterols, terpenoids and terpenes) monitored by GC-MS. This was not the case for XXIV-67 (a small flake), however, and the GC-MS results for this artefact are discussed further in the next section.

Of the 43 artefacts with no traces of use recorded in the initial inspection, 20 were interpreted as used after use-wear analysis; possible use (n = 7), probable use (n = 5) and definite use (n = 8) (Table 1). Biomarkers for either cholesterol, abietic acid or β-sitosterol were detected on six artefacts with no traces of use found in the initial inspection; however, three of these revealed traces of use after use-wear analysis: XXV-4203 (possible use), XXVI-4965 (possible use) and XXVI-4966-A (definite use); and three did not: XXIV-79, XXVI-4531 and XXVI-5048. The associated GC-MS results are discussed in the next section.

### GC-MS biomarker analysis: residue source identification using parent and proxy molecules

The targeted compounds, and their provenance, are listed in Table S2. A list of analytes is also included in the spreadsheet containing the complete quantitative GC-MS dataset (Supplementary Information).

To screen for contamination originating from the laboratory and the sedimentary background, two types of control samples were analysed with the artefact solvent extracts. Reagent blank control samples were prepared by aliquoting the extraction solvent (used to extract the artefacts) into vials and processing them in the same manner as with the artefact extracts. Contamination that is introduced in the laboratory during the sample preparation process will be evident in the reagent blank data. The chemical profiles of artefacts with no traces of use were utilised as baseline data to monitor the analyte contributions from the depositional environment. The analysis of sediment found around archaeological objects to screen for environmental contamination has been suggested, but the extraction efficiency is different between stone and sediment matrices. Unused stone artefacts are a more comparable matrix to stone tools, and so were used instead of sediment as a control for environmental contamination. For the purposes of providing sedimentary ‘baseline’ data, the ‘background’ chemical profile consists of C_10_–C_20_ saturated fatty acids, with even-carbon numbered fatty acids found at significantly greater abundances than the odd-carbon numbered fatty acids^7^. Odd-chain saturated fatty acids of chain length ≥ C_17_ were detected infrequently (on six artefacts classified as unused by microscopy after artefact cleaning; Tables 1 and S1) and below the limits of quantification of the analytes. No sterols and terpenoids were found on the artefacts that were classified as unused after cleaning, except for three cases (not including artefact XXIV-67, which is an exceptional case and will be discussed separately). In these three cases, only one sterol or terpenoid was found, rather than a combination of taxonomically-specific analytes. Saturated dicarboxylic acids and mono-hydroxy acids were not detected. Pinene and limonene were consistently detected on all artefacts and so these compounds are likely contributed by the sediment (although other sources cannot be excluded) and not considered useful for residue source determination. Other terpenes were not detected in the ‘background’ chemical profiles.

Lipids such as fatty acids are ubiquitous and are found in both the sediment and in rocks due to natural processes. In general, even-carbon numbered fatty acids are present at higher abundances than odd-carbon numbered fatty acids^23^. Since the residues in this study are found on stone and exposed to sediment, the use of carbon number distribution of the full range of fatty acids and fatty acid ratios for the determination of residue sources are not meaningful, and were not used to determine the origin of the residues. However, the quantities of long-chained saturated fatty acids and analogues, sterols, terpenoids and terpenes that were extracted from the artefacts with traces of use found in the initial inspection are shown in Tables 2 and 3.

**Table 2:** Artefacts with attached plant residues identified from the presence of plant-derived analytes detected by GC-MS/MS.

Artefact	Long-chain saturated fatty acids and analogues	Sterols	Terpenoids
XXV-3931^a^	Tridecanoic (detectable^c^), pentadecanoic (55 ng) and heptadecanoic (detectable^c^) acids were found on the edge of interest, but not anywhere else on the artefact. Camphor was found (detectable^c^). Although there was a lack of the more taxonomically specific analytes present, the saturated C_10_–C_26_ alkanes profiles of the edge of interest and sedimentary ‘baseline' are significantly different, indicative of a plant residue. This is confirmed by the presence of use-polish on the artefact (Fig. 4).		
XXV-3956^a^	β-Sitosterol (detectable^c^), heptadecanoic (detectable^c^) and docosanoic acids (detectable^c^) were detected in the extract from the edge with traces of use, but not elsewhere on the artefact.		
XXVI-4257	12-hydroxydodecanoic acid (detectable^c^)	β-sitosterol (detectable^c^)	—
XXVI-4411^a^	Campesterol (235 ng) was found in the extract from the edge with traces of use. Oleic acid–TMS (library score = 94.38) was also detected in this extract after retrospective analysis of the full-scan data.		
XXVI-4412	—	β-sitosterol (detectable^c^)	—
XXVI-4533-A	Tricosanoic (326 ng), tetracosanoic (771 ng) and 12-hydroxydodecanoic acids (detectable^c^)	β-sitosterol (detectable^c^)	Abietic (4 ng) and ursolic (detectable^c^) acids
XXVI-4533-B	tetracosanoic acid (59 ng)	β-sitosterol (detectable^c^)	—
XXVI-4579	tetracosanoic acid (53 ng)	β-sitosterol (detectable^c^)	—
XXVI-4583	—	β-sitosterol (detectable^c^)	—
XXVI-5048^b^	—	β-sitosterol (12 µg)	—
XXVI-4965	—	—	Abietic acid (782 ng)
XXVI-4531^b^	—	—	Abietic acid (238 ng)

^a^GC-MS/MS data obtained from previous studies^7,8^.

^b^Interpreted as unused after use-wear analysis.

^c^Analyte concentration in the extract was between the limits of detection and quantification.

**Table 3:** Artefacts with attached plant and animal residues identified from the presence of plant- and animal-derived analytes detected by GC-MS/MS.

Artefact	Long-chain saturated fatty acids and analogues	Sterols	Terpenoids	Terpenes
XXIV-68	Tricosanoic (29 ng) and tetracosanoic (165 ng) acids	Cholesterol (15 ng) and β-sitosterol (detectable^c^)	Abietic (3 ng) and ursolic (detectable^c^) acids	—
XXIV-67^a,b^	Cholesterol (17 ng), oleanolic acid (350 ng) and ursolic acid (28 ng) were observed, as well as betulinic acid at detectable levels. Azelaic (detectable^c^), sebacic (134 ng) and thapsic (138 ng) acids were found, as were 12-hydroxydodecanoic (detectable^c^) and 16-hydroxyhexadecanoic (detectable^c^) acids. A suite of saturated fatty acids from pentadecanoic acid (56 ng) to tetracosanoic acid (34 ng) was also detected.			
XXV-3932	Tetracosanoic (626 ng) and azelaic (detectable^c^) acids	Cholesterol (45 ng) and β-sitosterol (detectable^c^)	—	—
XXV-3955	Tetracosanoic acid (99 ng)	Cholesterol (207 ng) and β-sitosterol (detectable^c^)	—	—
XXV-3953	Azelaic acid (detectable^c^)	Cholesterol (63 ng), campesterol and β-sitosterol (detectable^c^)	—	Camphor (46 ng)
XXV-3954^b^	Cholesterol (100 ng), abietic acid (37 ng), β-sitosterol (detectable^c^), tridecanoic (detectable^c^), nonadecanoic (detectable^c^), eicosanoic (556 ng), docosanoic (detectable^c^) and azelaic (detectable^c^) acids were found. Camphor (43 ng) was extracted from the artefact.			
XXV-4025^b^	Cholesterol (1.4 µg) was extracted from this artefact. β-Sitosterol (detectable^c^), tridecanoic (detectable^c^) and heptadecanoic (detectable^c^) acids were also detected.			
XXV-4619	Tetracosanoic acid (1 µg)	Cholesterol (219 ng) and β-sitosterol (detectable^c^)	—	—
XXVI-4414^b^	Cholesterol (32 ng), β-sitosterol (detectable^c^) and stigmasterol (detectable^c^) were detected, as well as heptadecanoic (detectable^c^), nonadecanoic (detectable^c^) and eicosanoic (545 ng) acids.			
XXVI-4532	—	Cholesterol (553 ng)	Oleanolic acid (890 ng)	—

^a^Intepreted as unused after use-wear analysis.

^b^GC-MS/MS data obtained from previous studies^7,8^.

^c^Analyte concentration in the extract was between the limits of detection and quantification.

Targeted GC-MS data were used to propose potential sources for the residues, while also ruling out other possible sources. The latter were aided by retrospective analysis of the full-scan GC-MS data, where peaks in the total ion chromatogram (TIC) (acquired in full-scan mode) of potentially archaeologically-significant extracts were investigated for each artefact. The Wiley 7n library was used to determine if there were potential matches to reference spectra, despite the limited selection of compounds associated with natural products (particularly in silylated form) found in standard mass spectral libraries^24^. The extracted ion chromatogram (EIC) function was also applied to the full-scan data to extract the masses of lipids and lipid analogues that are useful for source identification or indicators of chemical processes, but that were not incorporated into the targeted GC-MS methodology. The masses corresponded to the molecular ion and/or trimethylsilyl (TMS) derivative of the compound of interest. We were cautious of potential false negatives associated with the EIC data, as the absence of a molecular ion (particularly for the smaller molecules) may be a result of the hard ionisation mechanism employed by GC-MS (i.e., electron ionisation mass spectrometry, EI-MS) and not necessarily due to the absence of an analyte of interest in the sample.

#### Plant origin

**Figure 3:**
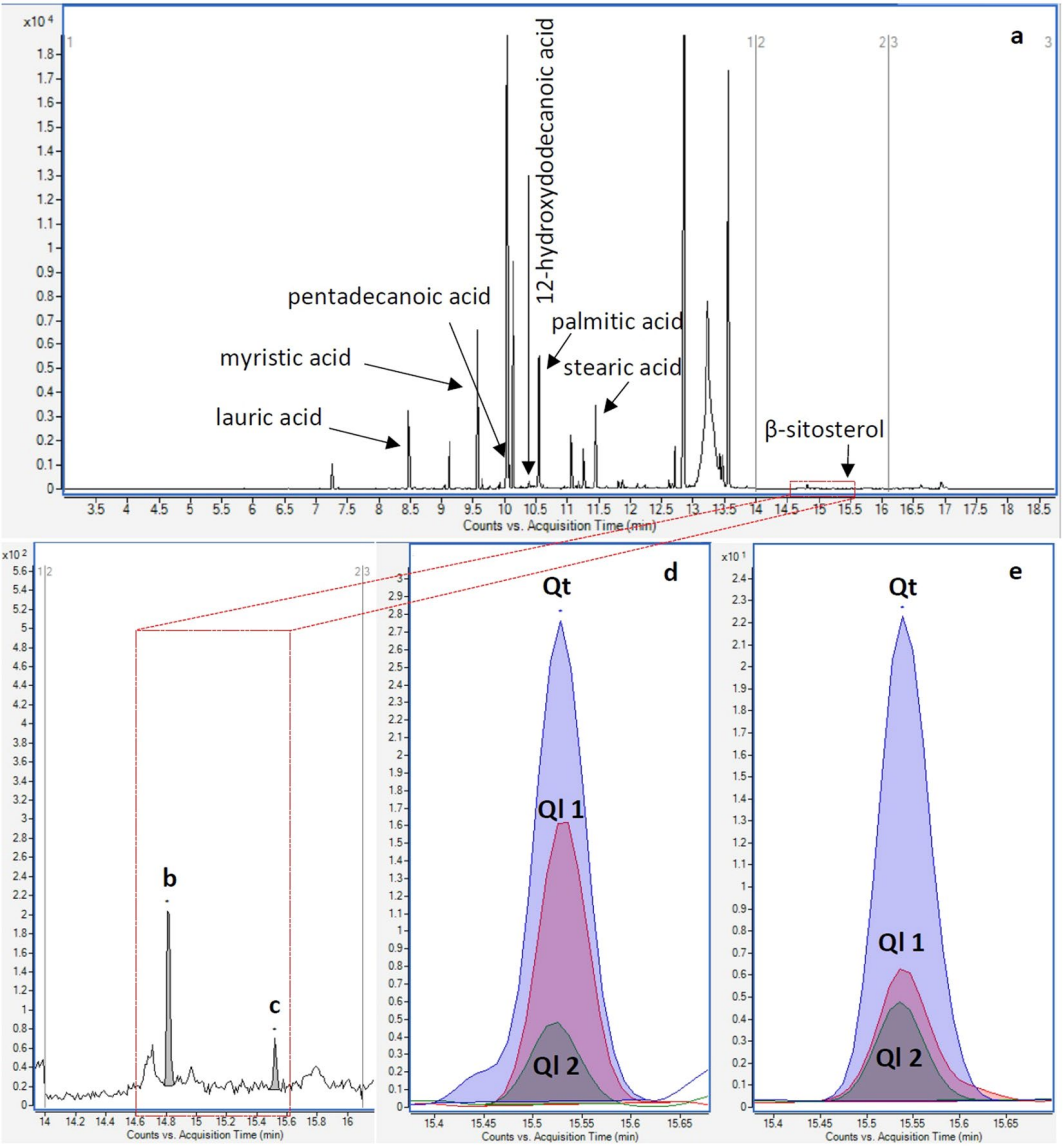
(**a**) TIC trace of the extract obtained from the edge of interest on artefact XXVI-4257, showing the elution of (**b**) cholesterol-D_7_ (internal standard) and (**c**) β-sitosterol within the sterol analogues time segment window (14.0–16.1 min). The positive identification of β-sitosterol was based on the corresponding elution times and MRM quantitative and qualitative ion transitions between (**d**) the analyte and (**e**) the β-sitosterol reference standard. Notes: Qt = quantitative ion; Ql = qualitative ion; X-axis = retention (acquisition) time (min); Y-axis = analyte abundance (counts). The unlabelled peaks in the TIC trace (**a**) are unidentified compounds and were not analytes targeted in this study.

In MRM data acquisition, the ions that are not of interest are not monitored; only data for characteristic ion transitions that are observed for the analytes of interest are collected. Typical full-scan mass spectra that are obtained using single quadrupole MS, and contain all peaks within the *m/z* range set by the analyst, are not collected in this mode of acquisition. Therefore, analyte identification using MRM data (i.e., the comparison of quantitative and qualitative ion transition peaks expected for each compound) is less affected by contamination and noise, and is more sensitive and specific than the use of full-scan mass spectra. The analyte identification procedure was executed for 86 lipids, terpenes, terpenoids, alkanes and their analogues potentially found on each of the 69 analysed stones (a total of 100 samples including both the edge(s) of interest and total submersion extracts). Due to the copious volume of data generated, quantitative processing methods were developed to automate analyte peak detection and integration using vendor software for high-throughput quantification and review of the data. An example of a positive identification is presented in Fig. 3, showing the TIC trace (Fig. 3a) as well as the retention time, quantitative and qualitative MRM transition data used to positively identify β-sitosterol (diagnostic of plant material) on artefact XXVI-4257. Two sterols were found in the time window assigned to the sterol analytes: the deuterated cholesterol internal standard (Fig. 3b) and the analyte of interest (Fig. 3c). The retention time of the latter analyte was 15.528 min (Fig. 3d), corresponding to 15.536 min observed for the β-sitosterol reference standard (within the ± 0.1 min threshold set by the method; Fig. 3e). Also, the quantitative ion transition (Qt = 357 − > 95) and the two qualitative ion transitions (Ql 1 = 396 − > 381 and Ql 2 = 396 − > 367) expected for β-sitosterol were observed for the analyte of interest, and in the expected ratios (within relative response uncertainties of ± 30% and ± 20% for Ql 1/Qt and Ql 2/Qt ratios, respectively). Positive identifications of analytes of probable archaeological significance (determined after comparison with the reagent blank and sediment ‘baseline’ control data) are summarised in Tables 2 and 3. For the complete quantitative GC-MS dataset, please refer to the spreadsheet submitted as part of the Supplementary Information.

Lipid residues of plant origin possess a higher composition of mono-, di- and tri-unsaturated C_18_ fatty acids than in non-plant sources. These unsaturated molecules are susceptible to oxidative processes, however, decreasing their likelihood of detection in ancient residues^25^. The use of dihydroxy acid derivatives, such as 11,12-dihydroxyeicosanoic acid and 13,14-dihydroxydocosanoic acid^26–28^, have been used as proxy molecules for the unsaturated parent compounds. Dihydroxy acids are more useful, and more likely encountered, than w-(o-alkylphenyl) alkanoic acids (cyclic proxy molecules derived from the heating of unsaturated fatty acids), because the oxidation reaction can occur at room temperature^29^. In addition, the C_11_-C_13_ (and longer chain) dicarboxylic acids are also of interest, as they are mainly encountered in plant waxes^26^, as are longer-chained fatty acids (typically greater than 20 carbon atoms)^30^. EICs of the TMS derivatives of 11,12-dihydroxyeicosanoic acid (*m/z* 561), 13,14-dihydroxydocosanoic acid (*m/z* 589) and the C_11_-C_13_ dicarboxylic acids (*m/z* 360, 374 and 388, respectively) had no peaks of interest. However, C_21_-C_26_ saturated fatty acids were found on 14 of the artefacts (13 of which were interpreted as used), and nine contained quantifiable concentrations (in the extracts) of tetracosanoic acid (C24:0). Tricosanoic acid (C23:0) was also quantified for three of the latter artefacts: XXVI-4533-A, XXIV-68 and XXIV-67 (Tables 2 and 3). These compounds were not found on any of the other artefacts. Similarly, azelaic acid (C_9_ dicarboxylic acid), which is associated with the ‘drying reaction’ of plant oils^5^, was detected in residues on artefacts XXV-3932, XXIV-67, XXV-3954, XXVI-4411 and XXV-3953 (Tables 2 and 3).

Overall, 22 artefacts show evidence of plant material, based on the identification of phytosterols, terpenoids and camphor, which is a terpene sourced from plants commonly found in Asia, including Indonesia (Tables 2 and 3). The later use-wear results indicate the presence of use-wear on 19 of these 22 artefacts, with confidence ranging from possible to definite use (Tables 1–3 and S1). The identification of long-chain fatty acid lipids, in addition to the more taxonomically-specific analytes, in most of the 19 residues increases the probability that these are indeed related to tool use. These findings do not apply to XXVI-5048 (a small longitudinal split flake), XXVI-4531 (a ‘retouched’ split cobble) and XXIV-67 (a small flake). Use-wear analysis indicated that these artefacts were unused, and only either β-sitosterol or abietic acid was found on the former two artefacts; no acidic lipids of archaeological interest were found (Table 2). As such, the use of XXVI-5048 and XXVI-4531 for resource processing is unlikely, based on the absences of distinctive use-wear and of other biomarkers. The plant biomarkers detected on these artefacts may derive from leaching of neighbouring floral material; for example, β-sitosterol was found at an anomalously higher concentration on XXVI-5048 compared to the other artefacts. In the case of artefact XXIV-67, a suite of organic biomarkers consistent with plant and animal residues were detected, but use-wear analysis indicated no traces of use (discussed further in the section titled *Degree of agreement between GC-MS multiresidue analysis and use-wear analysis*).

**Figure 4:**
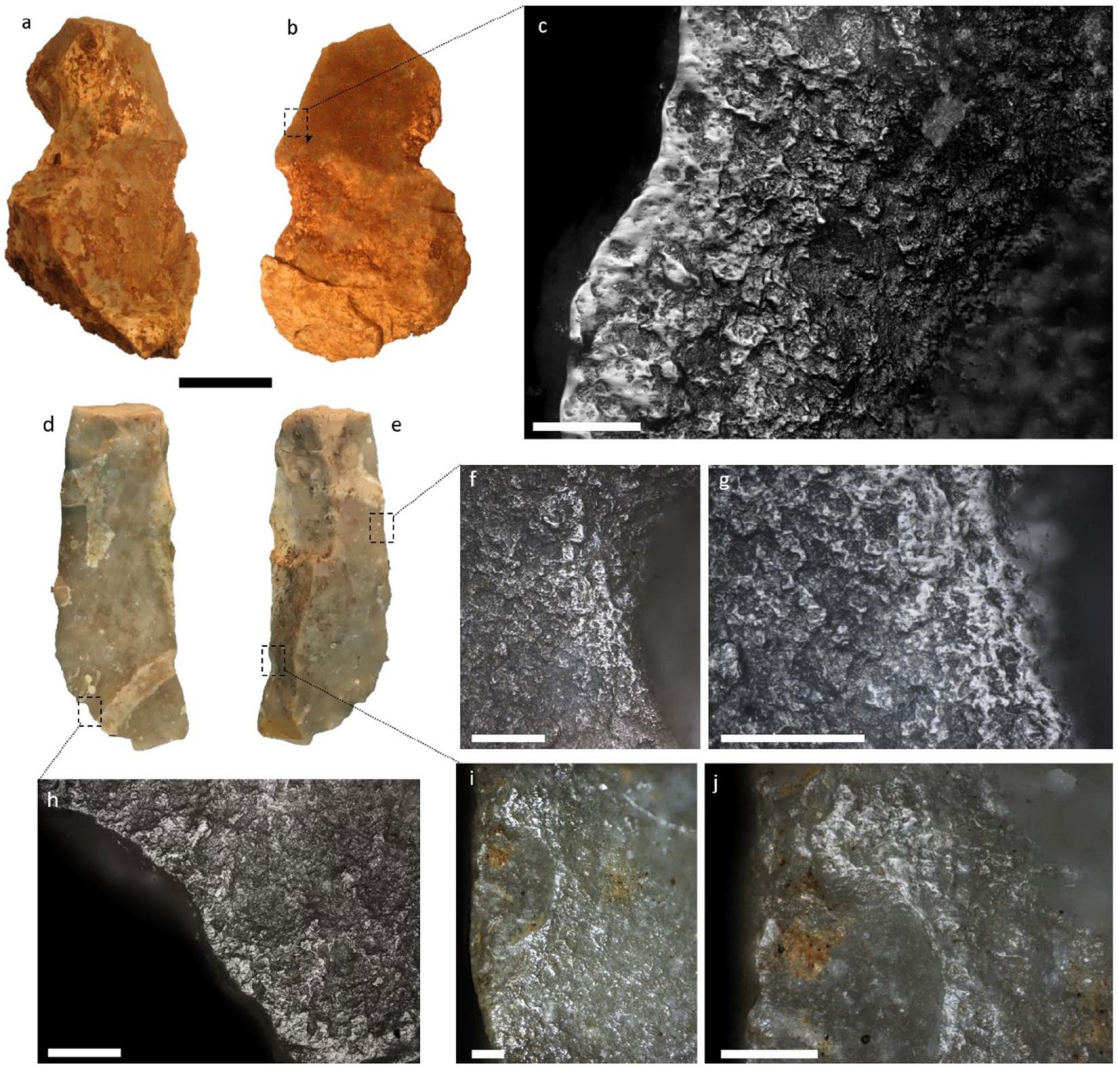
Use-wear documented on artefacts XXV-3931 and XXVI-4257: (**a**,**b**) ventral and dorsal views of XXV-3931, showing attached sediment before cleaning, (**c**) use-polish (after cleaning) on right lateral edge (dorsal) of XXV-3931 characteristic of working siliceous plants; note the highly connected zones of polish and the distinct boundary between the polish and the non-polished zones, (**d**,**e**) ventral and dorsal views of XXVI-4257 after cleaning, (**f**,**g**) negative flake scar and use-polish on the right lateral edge (dorsal) of XXVI-4257 characteristic of plant working, (**h**) use-polish on the left lateral edge (ventral) of XXVI-4257, (**i**,**j**) negative flake scar and use-polish on the right lateral edge (dorsal) of XXVI-4257. Scale bar = 2 cm (**a**,**b** and **d**,**e**) and 100 µm (**c** and **f**–**j**). Each micrograph is from stacked images, captured manually on the Olympus metallographic microscope (model BX51) at different focal depths.

Worked material can sometimes be recognised from diagnostic use-wear traces on the used edge(s) of a stone tool. At least two of the artefacts in which plant residues were identified with GC-MS (XXV-3931 and XXVI-4257) also displayed well-developed use-wear traces suggesting the working of plant materials (Fig. 4). Wear on XXV-3931 was characterised by a bright, well-connected use-polish diagnostic of working siliceous plant materials. The sharp boundary between the polished and non-polished zones of the tool edge indicates contact with a relatively hard plant tissue, as found in bamboo or rattan. Wear on XXVI-4257 is also consistent with the processing of plant materials, with a well-developed, undulating, highly connected use-polish on three of the tool edges. The presence of such wear traces further supports that the residues detected on these tools are related to use.

**Figure 5:**
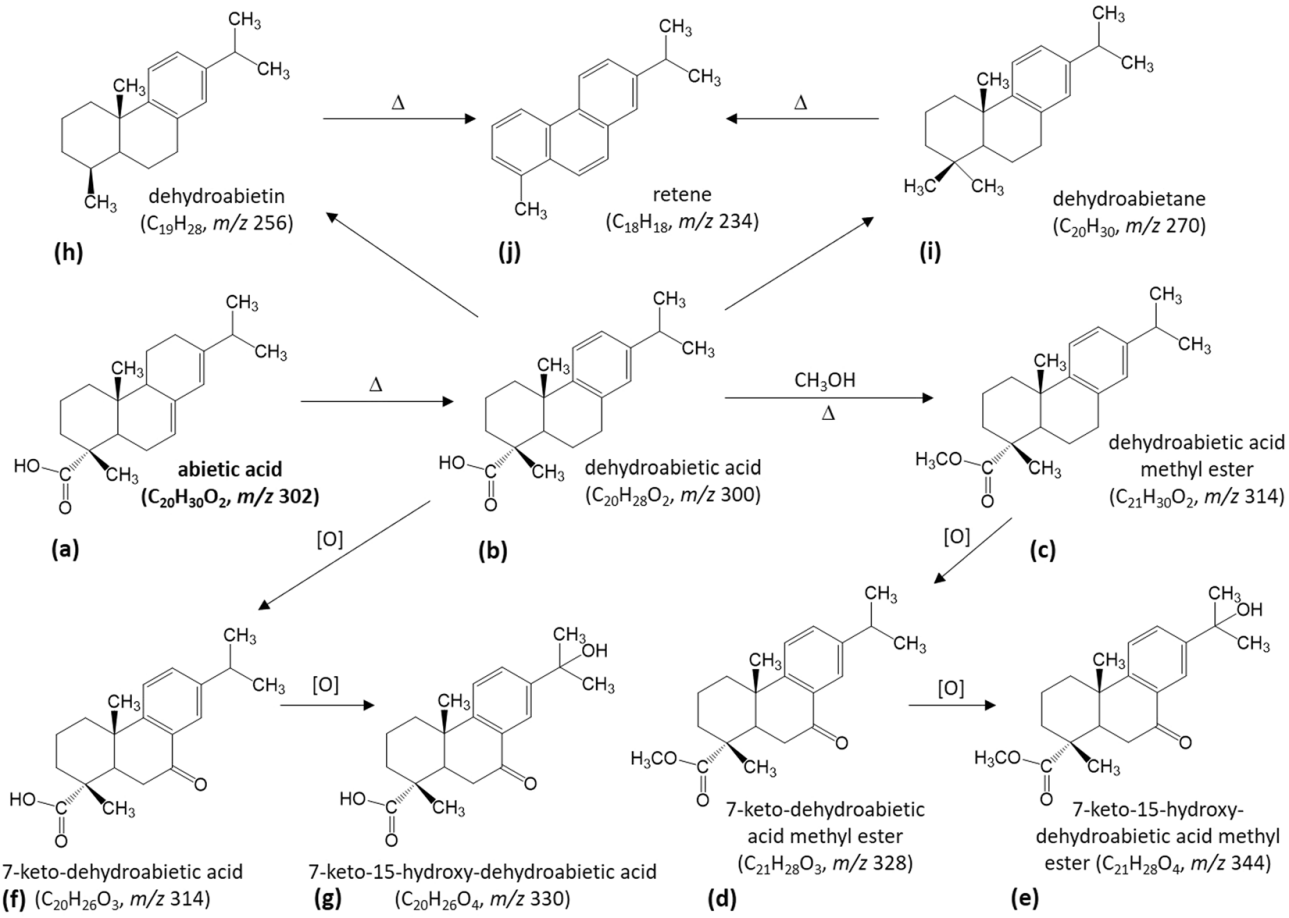
Reaction scheme outlining the conversion of abietic acid (**a**) upon heat treatment. Subsequent to dehydrogenation, dehydroabietic acid (**b**) is produced. In the presence of wood from plants containing methanol and further heating, dehydroabietic acid undergoes esterification with methanol to form dehydroabietic acid methyl ester. (**c**) Keto- and hydroxy- analogues of this methyl ester compound—(**d**,**e**), respectively—are formed through oxidation reactions under aerobic conditions. Dehydroabietic acid can also undergo these reactions to form similar oxidation products (**f**,**g**). Alternative transformation pathways involve decarboxylation of dehydroabietic acid to produce dehydroabietin (**h**), or modification of its carboxylic acid group to form dehydroabietane (**i**). Further exposure to high-temperature heating (at temperatures associated with boiling for pitch) results in the aromatisation of these reaction products to form retene (**j**), the final and most stable reaction product. These transformation products, along with abietic acid, can be used as indicators of heat-related processes involving plant materials containing abietic acid. The *m/z* values of these compounds were calculated and retrospectively extracted (either as the native species or as the TMS derivative) from the non-targeted GC-MS data collected in this study.

Much attention has been given to the plant-derived diterpenoid abietic acid and its reaction products following preparation of resin from the Pinaceae family to produce pine pitch^1,26,31–34^. These conversions are thermally induced, so the presence of these compounds indicates the use of heat (Fig. 5). At Liang Bua, approximately 18% of the modern human stone artefact assemblage shows signs of exposure to heat^20^. Since abietic acid was found in the solvent extracts corresponding to six artefacts (XXIV-67, XXIV-68, XXV-3954, XXVI-4531, XXVI-4533A and XXVI-4965), and hearth and hearth-like features are associated with anatomically modern humans at Liang Bua^35^, we extracted EICs of the masses of these abietic acid transformation products (as the TMS derivative or native species (*m/z* values in Fig. 5), where appropriate) from the full-scan data. No evidence of the presence of these compounds was found, and the lupeol and betulin triterpenoids were not detected in any of the samples. This result is not surprising because betulin, in particular, is associated with the bark of birch trees^36^ that are widespread in the northern hemisphere. Nevertheless, these two analytes were integrated into our analytical workflow, because a major priority was to be able to apply the same targeted MS/MS methodology to analyse artefacts recovered from different sites, and from diverse geographical regions, in future studies.

#### Animal origin

Cholesterol was used to identify artefacts with deposited residues of animal origin and was found on 15 artefacts, either with plant material (Table 3) or without (Table 4). In the case of artefact XXV-4256, cholesterol was found on the artefact, but not on the edge with traces of use, as determined by the initial inspection. Based on the use-wear analysis, it is placed in the ‘probable use’ category (Tables 1 and S1). Whether the cholesterol deposition was a result of tool use related to animal processing, or was due to leaching from neighbouring organic material, requires further investigation. It is more difficult to ascertain the sources of animal-derived than plant-derived materials, because there are fewer taxonomically-specific compounds directly derived from animals and amenable to GC-MS that can be used for unequivocal identification.

**Table 4:** Artefacts with attached animal residues identified from the presence of cholesterol detected by GC-MS.

Artefact	Cholesterol concentration in the solvent extract (ng/mL)	Solvent extract
XXV-4256	70	E2^b^
XXVI-5002	24	E1-1^c^
	30	E1-2^c^
XXV-4203	42	E3^d^
XXVI-4966-A	22	E3^d^
XXIV-79^a^	34	E3^d^

^a^Interpreted as unused after use-wear analysis.

^b^Extract from total submersion of the artefact, after localised extraction from the edges with traces of use (determined by the initial inspection).

^c^Extract from localised extraction of the edges of the artefact.

^d^Extract from total submersion of the artefact with no traces of use (determined by the initial inspection).

The palmitic acid/stearic acid (P/S or C_16_:C_18_) ratio has been used previously to determine whether a residue is of plant or animal origin^28,37,38^. The P/S ratio was not used in our study, however, for two reasons. First, fatty acids oxidise at different rates^2,39^ and the rate is affected by the environmental context. Second, the residues studied here may have multiple sources, as well as sedimentary contributions of palmitic and stearic acids. The use of the C_16_:C_18_ ratio is more valid in cases where the residues are not directly exposed to the sedimentary environment (with ceramics, for example, where the residues are trapped within the ceramic matrix and somewhat protected). Significant quantities of odd-chain fatty acids can be indicative of ruminant lipids; we used this indicator with caution, since these compounds were also detected in the solvent extracts from unused artefacts. Odd-chain fatty acids were detected more frequently among the potentially- and definitely-used artefacts, but not at significantly higher concentrations than the even-chain fatty acids, which argues against the presence of ruminant lipids. This result is not surprising, since ruminant mammals such as deer and cattle are only observed within the past ~2,000 to 3,000 years of the Liang Bua stratigraphic sequence^17^.

#### Aquatic origin

Retrospective (i.e., EIC) analysis of the full-scan data demonstrated that aquatic biomarkers are absent in the recovered residues. The TMS derivatives of the w-(o-alkylphenyl) alkanoic acids, dihydroxy acids, isoprenoid fatty acids and monosaturated fatty acids were used as aquatic biomarkers (see Supplementary Information for further details). Although this is a negative result, it is still worth mentioning as it is consistent with Liang Bua biostratigraphy, which shows low levels of aquatic shells in the stratigraphic units from which almost all of these artefacts were recovered^17^.

#### Summary of the types of residues found on the artefacts

Sterols, terpenoids and terpenes derived from plant or animal origin were found on 27 of the 69 analysed stones examined in this study (Tables 2–4). Use-wear analysis confirmed traces of use on 23 of these 27 artefacts (Tables 1 and S1). No traces of use were found on the remaining four artefacts; of these, two (XXVI-4531 and XXVI-5048) were likely not used, based on the combination of no evidence of use-wear and the detection of only one plant biomarker on each artefact; one (XXIV-67) possessed a compelling suite of plant and animal biomarkers; and the other artefact (XXIV-79) presented evidence of animal residues. These 27 chemical residue profiles were categorised into three groups based on the probable origin(s) of the residues: (i) plant-only (n = 12), (ii) animal-only (n = 5) and (iii) plant and animal (n = 10).

For the 42 artefacts where no sterols, terpenoids and terpenes were detected, chemometric analysis of the data are needed to further evaluate the influence of the saturated fatty acids and alkanes on the overall chemical profiles. Although these analytes are not very taxonomically-specific by themselves, their relative abundances may enable us to distinguish between artefacts that were genuinely not used from those that were. In this study, however, the taxonomically-specific analytes were not detected, due to either poor residue preservation or because no residues, or only a limited quantity of them, ever adhered to the artefact when it was used.

#### Degree of agreement between GC-MS multiresidue analysis and use-wear analysis

There was general agreement between the GC-MS and later use-wear results for 47 of the 69 analysed stones, but some discrepancies were observed. It is important to note that some discrepancies are to be expected. However, as with all scientific processes, all potential reasons for inconsistencies, beyond those to be expected, should be explored and considered. Some artefacts had use-wear, such as artefacts XXV-4255, XXV-4443 and XXVI-4534-A (Table 1), but these proved relatively unremarkable from a biomarker perspective due to the absence of taxonomically-specific analytes such as sterols, terpenes and terpenoids. On 20 artefacts, the use-wear analysis identified traces of use that were not identified in the initial macroscopic/low-magnification inspection (Table 1). However, only three of these artefacts (XXVI-4965, XXV-4203 and XXVI-4966-A) had corresponding GC-MS evidence indicating the presence of taxonomically-specific biomarkers that agreed with the use-wear analysis. Potential reasons for these inconsistencies are that the organic biomarkers have not persisted through time to be detected or the residues are simply not detectable by GC-MS, due to either general molecule/instrument incompatibilities or unsuitability with the specific low molecular weight organic biomarker methodologies adopted in this study. In other cases, GC-MS molecular evidence for the presence of use-residues was convincing, but no or minimal evidence of use was documented in the use-wear analysis. For example, biomarkers consistent with plant and animal origins that were uncommonly observed in the surrounding sediments were extracted from artefact XXIV-67, but the use-wear analysis indicated no traces of use. Perhaps this stone artefact was not used enough to develop any diagnostic use-wear patterns, or perhaps it came into incidental contact with plant and animal tissue but was never used. However, chemometric analysis (multivariate statistical analysis of chemical data) is one investigative pathway that may help further explain the observed discrepancies between the microscopy/use-wear and GC-MS data sets. The incorporation of complementary analytical techniques, such as Raman spectroscopy or Fourier transform infrared spectroscopy (FTIR), into the current workflow is another avenue worth exploring. Other artefacts with definite traces of use lacked GC-MS molecular evidence for the presence of use-residues, because their use was not linked with organic tissue but with pounding of minerals or pigment (XXV-3951) or flaking stone (XXVI-4655).

### Implications for residue preservation in the humid tropics

The presence/absence of particular types of biomarkers observed for the stone artefacts in this study are broadly consistent with expectations, considering the tropical setting of Liang Bua. The lack of sterol and stanol oxidation products, such as 7-ketocholesterol and 25-hydroxycholesterol, is not surprising, given the combination of percolating groundwater and typically humid conditions, and the degradation propensity of these compounds relative to saturated fatty acids and terpenoids. These types of compounds are more likely to be preserved on artefacts deposited in arid environments^11^. The clay content in the sediments surrounding the artefacts may also act as a catalysing agent^40^, facilitating the chemical transformation of the organic biomarkers of interest through various degradation pathways.

Despite the generally unfavourable conditions for organic biomarker stability at Liang Bua, the biomarkers that we have detected on the potentially-used edges of stone artefacts demonstrate that some organic compounds can be preserved for several thousands of years, including parent compound sterols such as cholesterol and β-sitosterol. As alternating wetting and drying or heating and cooling are typically not conducive to residue survival^4^, presumably such events did not occur to a great extent at Liang Bua. Preservation may, instead, have been assisted by conditions of relatively constant humidity and temperature. The presence of azelaic acid is indicative of the reaction of oleic acid involving the site of unsaturation on the molecule. The production of azelaic acid can occur through various chemical reactions, of which autoxidation is the most likely pathway on a stone artefact. Oxidation of oleic acid and cleavage of the carbon-carbon double bond was most likely carried out via a radical reaction with hydroperoxide intermediates^11^, which implies aerobic conditions when this occurred.

The identification of plant and animal biomarkers on stone artefacts at Liang Bua suggests, therefore, that certain organic compounds can be retained for several millennia under tropical conditions—provided that the depositional context of the artefacts (i.e., the chemical conditions of the surrounding sediments and the influence of environmental factors such as moisture and temperature) is conducive for residue preservation.

### Resource use by anatomically modern humans at Liang Bua

Use-wear analysis indicates that 18 artefacts have definite traces of use, and GC-MS profiles suggest that two of these were used for processing animal tissue, five were used for processing plants and three were used on both plant and animal tissue. Use-wear analysis indicates that an additional 25 artefacts have possible or probable traces of use, and of these GC-MS profiles suggest that two were used for processing animal tissue, five used for processing plants and seven were used on both plant and animal tissue. We have provided various explanations as to why the analysed GC-MS profiles occur on some artefacts with no traces of use and why they are absent on some artefacts that do have clear traces of use. Further analyses of the use-wear and GC-MS results are needed, particularly to model presence/absence of use-wear forms and residue types. Based on the GC-MS findings, animal residues (with and without plant residues) were observed for artefacts of various ages (11–5, 14.01–11.75 and 18.97–17.45 ka), whereas plant residues were only observed for those in the 14.01–11.75 and 18.97–17.45 ka time ranges. These observations are almost certainly due to our limited sample size. A larger sample size will allow spatial and chronological variation to be assessed.

More artefacts contained plant rather than animal residues, and different combinations of the taxonomically-specific analytes within each of the plant residues suggests that they originate from different sources (i.e., different plants). However, further taxonomic identification of the plants is difficult based on the GC-MS results, as the monitored organic biomarkers are not exclusive to any one plant. Examination of the siliceous polish and remnants (e.g., phytoliths and minerals) that were left behind by the resource-processing task, but not detected by GC-MS, may provide further insights into the material worked; this will be the focus of a separate use-wear study.

The raw material composition of the relatively small number of artefacts selected for our study is comparable to that found in the overall Late Pleistocene and Holocene assemblages at Liang Bua associated with anatomically modern humans^17,20^. In the sub-sample of artefacts selected for GC-MS analysis, the number of artefacts made of chert and silicified tuff are approximately equal (Table 5), as are the number of artefacts with residues within each respective type of raw material. Thus, no clear trend is observed in terms of observed residues and artefacts made of a particular raw material. Overall, the percentages of artefacts with residues compared to the total within each type of raw material (37–57%) are similar, with slightly higher evidence of use seen on artefacts made of less common raw materials (chalcedony and jasper). The frequency of residue detection by GC-MS within each technological class (32–86%) is more variable (Table 6). Residues were found more often on retouched flakes (86%) and broken retouched flakes (60%), which supports the idea that retouched flakes were used more intensively than unretouched flakes, and, in some cases, used until they broke (Table 6). Perhaps for the same reason, residues are slightly more common on broken flakes (38%) than on complete flakes (32%). However, these frequencies may not be statistically different from one another given the limited sample sizes. Another consideration is that the retouch process—resulting in ridges and steps—aids the preservation of residues.

**Table 5:** Number of artefacts with plant and/or animal residues, by stone raw material.

Stone raw material	Number of artefacts with residues^a^	Total number of analysed stones^b^	Artefacts with residues compared to the total (%)
Chalcedony	4	7	57
Chert	12	29	41
Silicified tuff	10	27	37
Jasper	1	2	50
Other^c^	0	4	0

^a^As determined by GC-MS organic biomarker analysis.

^b^Within the sub-sample selected for this study.

^c^Andesite (n = 2) and quartz (n = 2).

**Table 6:** Number of artefacts with plant and/or animal residues, by technological class.

Technological class	Number of artefacts with residues^a^	Total number of analysed stones^b^	Artefacts with residues compared to the total (%)
Broken flake	6	16	38
Retouched flake	6	7	86
Complete flake	10	31	32
Core/retouched split cobble	2	5	40
Broken retouched flake	3	5	60
Other^*c*^	0	5	0

^a^As determined by GC-MS organic biomarker analysis.

^b^Within the sub-sample selected for this study.

^c^Non-diagnostic artefact (n = 1), non-artefact (n = 1), broken pounding stone (n = 1), debris (n = 1) and broken hammerstone (n = 1).

## Conclusions and Future Work

In this methodological study, we have successfully identified non-volatile and volatile low molecular weight organic compounds on 69 stones (including 68 artefacts and one non-artefact) recovered from the archaeological site of Liang Bua in a tropical environmental setting. Potential use-residues were recovered from 27 artefacts and, based on the presence of sterols, terpenoids and terpenes, their potential sources were broadly grouped into one of three categories: plant-only, animal-only or a mixture of both. Comparison of the results from GC-MS and use-wear analyses indicates the potential of our methodology, which rapidly identifies chemical profiles of residues at low levels of detection. However, while there is reasonable agreement between the GC-MS results and conventional use-wear study, there remain conflicts and uncertainties in specific cases that suggest the need for further refinement and analyses of the relationships between use-wear and residues.

We are currently investigating chemometric analysis to determine the likely origin of residues recovered from prehistoric artefacts, by simultaneously examining all of the targeted biomarkers within the same residue, and to assess the influence of suites of saturated fatty acids and alkanes on the overall chemical profiles. Further refinement to determine residue origins with confidence is especially necessary when the chemical and environmental conditions of the deposits surrounding the artefacts are not the same for each artefact. Chemometric analysis of the multivariate GC-MS data may also allow better discrimination between artefact residues that are the result of resource processing rather than leaching from faunal or floral remains buried in the same or surrounding sediments. Such analyses could potentially establish linkages between similar residues based on the overall shapes of the chemical profiles, even though some of the more taxonomically-specific analytes are missing due to differential preservation. The use of chemometric analysis will also help capture the overall chemical profile of valid control samples at the site. Although control samples were innovatively included in this study, a greater number of controls should be included in future analyses to enable meaningful chemometric analysis. Finding samples that can function as suitable controls is difficult from a chemical perspective, as ‘ordinary’ stones subjected to the same environment as those with archaeological-significant residues are the best to use. This is because they undergo similar diagenesis and, if they are in a similar stratigraphic location, are potentially exposed to leaching of similar material. However, it can be difficult to determine whether a stone can validly be used as a control, as it is possible for artefacts to be used but not display any use-wear. Chemometric analysis will hopefully shed further light on this issue.

Finally, the use of actualistic experiments to further our understanding of archaeological residue preservation is also of interest. As part of our research, we have completed a series of taphonomic experiments focussing on (1) transfer of organic material through sediment and its persistence on stone flakes, and (2) lipid preservation and transformation pathways of various materials over time and under certain conditions. The outcomes of these experiments will help refine the interpretation of the results obtained in the present study from the perspective of residue source determination.

## Supplementary information

Supplementary Information

Supplementary Information

## Data Availability

The datasets generated and/or analysed during the current study are either included in this published article (and its Supplementary Information files) or are available from the corresponding author on reasonable request.
