# Supplementary material for: Combined organic biomarker and use-wear analyses of stone artefacts from Liang Bua, Flores, Indonesia
Source: Sci Rep. 2019 Nov 26;9:17553. doi: 10.1038/s41598-019-53782-2 (PMC6879511; doi:10.1038/s41598-019-53782-2)
Supplement: Supplementary file 1 — Supplementary Information [file 41598_2019_53782_MOESM1_ESM.pdf]

Table S1: Use-wear results for the Liang Bua artefacts analysed in this study. Edge scarring termination types: b, bending; f, feather; s, step; h, hinge; Striation orientation: =, parallel to edge; /, diagonal or perpendicular to edge; Location on flake: EPE, external platform edge; LL, left lateral; RL, right lateral. A use confidence score of zero refers to high confidence that the artefact has not been used. Use scores were assigned as: 1 = possible use; 2 = probable use; 3 = definite use, but unsure of the material processed; and 4 = definite use, and the material can be identified by use-wear. GC-MS profiles: P, plant; A, animal; B, both plant and animal.

Sector	Lithic #	Spit	Stratigraphic Unit	Age range (ka)	Artefact class	Stone material	Surface weathering	Cortex (%)	**GC-MS profiles**	**Use Confidence**	Polish stage	Edge scarring	Edge rounding	Striations	Location on flake	Mode of use	Material worked
XXIV	42	6	8	5–1	flake	silicified tuff	no	30	**0**	**3**	2	bs	low	/	EPE	scraping	cf. skin
XXIV	43	6	8	5–1	non-artefact	quartz	no	20	**0**	**0**	0	s	0	0	n/a	n/a	n/a
XXIV	56	7	8	5–1	broken retouched flake	silicified tuff	no	30	**0**	**3**	1	shfs	high	/	proximal	scraping	hard, cf. wood
											2	shfs	high	/	RL	scraping	hard cf. wood
XXIV	67	8	8	11–5	flake	silicified tuff	no	0	**B**	**0**	0	bs	0	0	n/a	n/a	n/a
XXIV	68	8	8	11–5	broken retouched flake	chert	no	0	**B**	**2**	1	fbs	low	/	distal end & sides	scraping	uncertain
XXIV	69	8	8	11–5	flake	chert	no	50	**0**	**3**	3	b	low	/	RL	scraping, slicing	siliceous plant
XXIV	79	6	8	11–5	broken flake	chalcedony	no	0	**A**	**0**	0	0	0	0	n/a	n/a	n/a
XXV	3923	50	6	14.01–11.75	flake	chert	no	0	**0**	**2**	2	0	low	=	RL	uncertain	uncertain
											1	f	low	0	RL	uncertain	uncertain
XXV	3931	51	6	14.01–11.75	flake	chalcedony	no	10	**P**	**4**	4	bs	high	/	LL	scraping & cutting	hard siliceous plant, cf. bamboo, rattan
XXV	3932	51	6	14.01–11.75	flake	chert	no	10	**B**	**1**	1	fbs	low	0	RL	n/a	uncertain

XXV	3950	52	6	14.01–11.75	core	chert	no	<5	**0**	**0**	0	0	0	0	n/a	n/a	n/a
XXV	3951	52	6	14.01–11.75	flake from pounding stone or anvil (no used flake margin)	andesite	no	100	**0**	**3**	1	0	0	0	dorsal surface	grinding stone/anvil	uncertain
XXV	3953	52	6	14.01–11.75	flake	silicified tuff	yes	1	**B**	**1**	1	bs	high	0	RL	uncertain	uncertain
											1	f	high	0	LL	uncertain	uncertain
XXV	3954	52	6	14.01–11.75	flake	chert	no	0	**B**	**4**	4	s	medium	/ =	RL	cutting, scraping	siliceous plant (hard?)
XXV	3955	52	6	14.01–11.75	flake	silicified tuff	yes	25	**B**	**1**	1	bs	low	0	LL	uncertain	n/a
XXV	3956	52	6	14.01–11.75	flake	chert	no	0	**P**	**1**	1	0	0	0	LL	uncertain	uncertain
XXV	3957	52	6	14.01–11.75	broken flake	silicified tuff	yes	100	**0**	**0**	1	fs	0	0	LL	n/a	n/a
XXV	3959	52	6	14.01–11.75	core	chert	no	10	**B**	**0**	0	0	0	0	n/a	n/a	n/a
XXV	4022	53	6	14.01–11.75	flake	silicified tuff	yes	60	**0**	**2**	1	bfs	low	=	RL	cutting?	uncertain
XXV	4025	53	6	14.01–11.75	broken retouched flake	silicified tuff	no	25	**B**	**2**	1	fs	low	0	RL	uncertain	uncertain
											1	fs	low	0	LL	uncertain	uncertain
XXV	4128	54	6	14.01–11.75	broken flake	chalcedony	yes	1	**0**	**0**	0	0	0	0	n/a	n/a	n/a
XXV	4130	54	6	14.01–11.75	broken flake	chert	no	10	**0**	**0**	b	b	0	0	n/a	n/a	n/a
XXV	4132	54	6	14.01–11.75	broken flake	chert	no	75	**0**	**0**	0	0	0	0	n/a	n/a	n/a
XXV	4203	55	6	14.01–11.75	retouched flake	chalcedony	no	50	**A**	**1**	0	fs	low	0	RL	uncertain	uncertain
XXV	4253	56	6	14.01–11.75	flake	chalcedony	no	0	**0**	**0**	0	0	0	0	n/a	n/a	n/a
XXV	4254	56	6	14.01–11.75	flake	silicified tuff	yes	25	**0**	**0**	0	b	0	0	n/a	n/a	n/a
XXV	4255	56	6	14.01–11.75	flake	chert	no	25	**0**	**3**	3	fs	high	0	RL	scraping	hard (cf. wood?)
											1	fs	high	/	LL	scraping	uncertain
											1	fs	high	0	dorsal ridge	uncertain	uncertain

XXV	4256	56	6	14.01–11.75	flake	silicified tuff	yes	5	**A**	**2**	1	s	high	0	RL	uncertain	uncertain
											1	s	medium	/	LL	scraping	uncertain
XXV	4283	57	6	14.01–11.75	flake	chert	no	40	**0**	**2**	1	fs	low	=	LL	cutting	soft
XXV	4286	57	6	14.01–11.75	flake	silicified tuff	yes	0	**0**	**0**	1	bs	0	=	RL	n/a	n/a
XXV	4443	62	6	18.58–13.75	broken flake	chert	yes	25	**0**	**3**	3	b	medium	=	distal	scrapping; cutting	plant
											2	f	medium	/	LL	uncertain	uncertain
XXV	4619	63	6	18.97–17.45	broken flake	chert	no	5	**B**	**2**	1	sf	low	0	?	uncertain	uncertain
											1	f	low	0	?	graving	
XXVI	4257	52	6	14.01–11.75	retouched flake	chert	no	5	**P**	**4**	4	sf	high	/=	LL	cutting & scraping	plant
											2	sf	medium	/=	distal	cutting & scraping	plant
											4	sf	sf	/=	RL	cutting & scraping	plant
XXVI	4337	53	6	14.01–11.75	retouched flake	jasper	yes	70	**0**	**0**	0	s	0	0	platform	n/a	n/a
XXVI	4338	53	6	14.01–11.75	distal flake	chert	no	5	**0**	**0**	0	0	0	0	n/a	n/a	n/a
XXVI	4410	54	6	14.01–11.75	core	chalcedony	no	20	**0**	**0**	0	0	0	0	n/a	n/a	n/a
XXVI	4411	54	6	14.01–11.75	retouched flake	chert	no	5	**P**	**3**	3	fs	low	=	LL	cutting	cf. plant
											3	fs	low	/=	RL	cutting & scraping	wood
XXVI	4412	54	6	14.01–11.75	broken flake	chert	no	50	**P**	**3**	2	fs	0	0	proximal break	uncertain	uncertain
XXVI	4413	54	6	14.01–11.75	flake	chert	yes	5	**0**	**2**	0	f	0	0	RL	scraping	uncertain
											0	fs	0	=	distal	cutting	uncertain
XXVI	4414	54	6	14.01–11.75	flake	chert	no	40	**B**	**1**	1	bs	0	=	LL	cutting	cf. plant
XXVI	4531	55	6	14.01–11.75	core	silicified tuff	yes	40	**P**	**0**	0	fs	0	0	platform	n/a	n/a
XXVI	4532	55	6	14.01–11.75	retouched flake	chert	yes	1	**B**	**3**	2	bs	low	/	LL	scraping	hard, wood?
											1	bfs	low	0	RL	uncertain	uncertain

XXVI	4533A	55	6	14.01–11.75	core	jasper	no	40	**P**	**1**	0	fs	low	0	platform	uncertain	n/a
XXVI	4533B	55	6	14.01–11.75	broken flake	silicified tuff	no	0	**P**	**3**	3	bsf	low	/ =	RL	scraping & cutting?	plant
											2	sf	low	0	distal	uncertain	uncertain
XXVI	4534A	55	6	14.01–11.75	flake	silicified tuff	no	0	**0**	**1**	1	bsf	low	0	distal	uncertain	soft
XXVI	4534B	55	6	14.01–11.75	broken flake	quartz	no	0	**0**	**0**	0	0	0	0	n/a	n/a	n/a
XXVI	4579	56	6	14.01–11.75	flake	chert	no	50	**P**	**1**	1	bs	low	0	RL	uncertain	uncertain
											1	bf	low	0	LL	uncertain	uncertain
XXVI	4581A	56	6	14.01–11.75	retouched flake	silicified tuff	no	0	**0**	**1**	1	f	low	0	distal	graving?	n/a
XXVI	4581B	56	6	14.01–11.75	flake	chert	yes	0	**0**	**0**	0	0	0	0	n/a	n/a	n/a
XXVI	4583	56	6	14.01–11.75	broken flake	chalcedony	?	0	**P**	**1**	1	b	low	0	LL distal	uncertain	uncertain
XXVI	4655	58	6	18.58–13.75	broken hammer/ pounding stone	andesite	yes	60	**0**	**3**	0	0	0	0	cortex	hammer or anvil	stone
XXVI	4828	63	6	18.58–13.75	distal flake	silicified tuff	no	50	**0**	**0**	0	0	0	0	n/a	n/a	n/a
XXVI	4963	62	6	18.58–17.45	flake	silicified tuff	yes	95	**0**	**0**	0	0	0	0	n/a	n/a	n/a
XXVI	4964	62	6	18.58–17.45	flake	silicified tuff	no	50	**0**	**2**	1	bs	low	0	distal	uncertain	uncertain
XXVI	4965	62	6	18.58–17.45	retouched flake	silicified tuff	yes	80	**P**	**1**	0	?	high	0	RL	uncertain	uncertain
											1	0	high	0	LL	uncertain	uncertain
XXVI	4966A	62	6	18.97–17.45	retouched flake	chert	no	50	**A**	**3**	2	s	low	/	distal	scraping	hard, wood? or bone?
XXVI	4966B	62	6	18.97–17.45	flake (retouch flake)	chert	no	0	**0**	**0**	0	0	0	0	n/a	n/a	n/a
XXVI	5002	63	6	18.97–17.45	flake	silicified tuff	no	5	**A**	**3**	1	bs	medium	0	RL	scraping	bone
											1	b	0	0	LL	uncertain	uncertain
XXVI	5004A	63	6	18.97–17.45	retouched flake	chert	no	90	**0**	**2**	1	?	low	/	proximal	scraping	uncertain

XXVI	5004B	63	6	18.97–17.45	flake	silicified tuff	no	0	**0**	**0**	0	0	0	0	n/a	uncertain	uncertain
XXVI	5045	64	6	18.58–17.45	flake	silicified tuff	no	70	**0**	**0**	0	0	0	0	n/a	n/a	n/a
XXVI	5046	64	6	18.58–17.45	retouched flake	chert	no	80	**0**	**3**	1	s	low	/	LL	scraping	uncertain
XXVI	5047	64	6	18.58–17.45	flake	chert	no	0	**0**	**0**	0	0	0	0	n/a	n/a	n/a
XXVI	5048	64	6	18.58–17.45	flake	silicified tuff	yes	0	**P**	**0**	0	0	0	0	n/a	n/a	n/a
XXVI	5106	65	6	18.58–17.45	flake	silicified tuff	no	50	**0**	**1**	0	s	low	0	LL	uncertain	uncertain
XXVI	5107	65	6	18.58–17.45	flake	silicified tuff	no	0	**0**	**0**	0	0	0	0	n/a	n/a	n/a
XXVI	5108	65	6	18.58–17.45	flake	silicified tuff	yes	50	**0**	**2**	1	bs	low	/	LL	scraping	uncertain
XXVI	5135	66	6	18.58–17.45	NDA	silicified tuff	yes	100	**0**	**1**	1	0	low	0	?	uncertain	uncertain
XXVI	5151	66	6	18.58–17.45	flake	chert	yes	0	**0**	**3**	1	s	low	0	LL proximal	cutting & scraping	uncertain, meat? (not plant)

Table S2: Compounds of interest targeted in the biomarker analysis, and their provenance.

compound	provenance
decanoic acid (C10:0)	Saturated fatty acids are stable compared to other biomarkers and are ideal for use in chemical investigations in the archaeological context. Although ubiquitous in nature, information on the combination and relative abundances of the fatty acids, and whether they are found alongside the other biomarkers of interest, is useful for narrowing down and potentially identifying the source of residues. Saturated fatty acids are found in both animals and plants in different abundances between animal and plant residues. Long-chain fatty acids (C_{20}–C_{30}) are found in significantly higher amounts in plant waxes (e.g. the surfaces of leaves and skins of fruit) than in animal fat or plant oil. Also, the presence of odd-chain fatty acids can be used as an indicator of bacterial activity typical of ruminant origin.
lauric acid (C12:0)	
tridecanoic acid (C13:0)	
myristic acid (C14:0)	
pentadecanoic acid (C15:0)	
palmitic acid (C16:0)	
heptadecanoic acid (C17:0)	
stearic acid (C18:0)	
nonadecanoic acid (C19:0)	
eicosanoic acid (C20:0)	
heneicosanoic acid (C21:0)	
docosanoic acid (C22:0)	
tricosanoic acid (C23:0)	
tetracosanoic acid (C24:0)	
hexacosanoic acid (C26:0)	
octacosanoic acid (C28:0)	
hentriacontanoic acid (C31:0)	
5α-cholestanol	Compound produced following the transformation of cholesterol in the environment. May potentially provide insights into the chemical environment surrounding the stone artefacts.
5-cholesten-3-one	An analogue of cholesterol. May potentially provide insights into the chemical environment surrounding the stone artefacts.
7-ketocholesterol	An analogue of cholesterol. May potentially provide insights into the chemical environment surrounding the stone artefacts.
7-dehydrocholesterol	An analogue of cholesterol. May potentially provide insights into the chemical environment surrounding the stone artefacts.
25-hydroxycholesterol	An analogue of cholesterol. May potentially provide insights into the chemical environment surrounding the stone artefacts.
β-sitosterol	Compound found in plants.
campesterol	Compound found in plants.
cholesterol	Compound found predominately in animals.
coprostanol	Compound resulting from the transformation of cholesterol in the gut of some animals. Often used as a biomarker for faecal matter.
ergosterol	Fungi-specific sterol. May potentially provide insights into the chemical environment surrounding the stone artefacts.
stigmasterol	Compound found in plants.
α-cholestanone	An analogue of cholesterol. May potentially provide insights into the chemical environment surrounding the stone artefacts.

abietic acid	Compound found in plants, particularly in resin derived from the Pinaceae family.
lupeol	Compound found in various plants.
betulin	Compound found in plants, commonly associated with the bark of birch tree.
oleanolic acid	Compound found in various plants.
betulinic acid	Compound found in various plants and is associated with betulin.
ursolic acid	Compound found in various plants.
azelaic acid	A proxy compound for unsaturated fatty acids that have undergone oxidation.
sebacic acid	A proxy compound for unsaturated fatty acids that have undergone oxidation.
thapsic acid	A proxy compound for unsaturated fatty acids that have undergone oxidation.
12-hydroxydodecanoic acid	A fatty acid analogue. May potentially provide insights into the chemical environment surrounding the stone artefacts.
16-hydroxyhexadecanoic acid	A fatty acid analogue. May potentially provide insights into the chemical environment surrounding the stone artefacts.
myrcene	Volatile compounds found in the fruit, leaves, skin and bark of a wide range of plant species, and may indicate the presence of resinous materials or wax.
limonene	
pinene	
g-terpinene	
squalene	
(-)-carvone	
(1S)-(-)-verbenone	
camphor	
linalool	
geraniol	
eucalyptol	
decane	Saturated alkanes can be used to identify and distinguish between different plants and plant components (e.g., epicuticular waxes).
dodecane	
tridecane	
tetradecane	
pentadecane	
hexadecane	
heptadecane	
octadecane	
nonadecane	
pristane	
eicosane	
phytane	
heneicosane	
docosane	
tricosane	
tetracosane	
pentacosane	
hexacosane	

heptaconsane	
octacosane	
nonacosane	
triacontane	
hentriacontane	
dotriacontane	
tritriacontane	
tetratriacontane	
pentatriacontane	
hexatriacontane	
heptatriacontane	
octatriacontane	
nonatriacontane	
tetracontane	

Aquatic biomarkers

To identify residues of aquatic origin, w-(o-alkylphenyl) alkanoic acids have been used for ceramic vessels[1-5]. Aquatic products contain C_{16}–C_{22} unsaturated fatty acids, which are converted to these cyclic compounds by heating to temperatures greater than 270°C. However, C_{18} w-(o-alkylphenyl) alkanoic acids are not specific to aquatic sources and can also be derived from plants and terrestrial animals. For heated artefacts, the more diagnostic markers for aquatic lipids are C_{20} and C_{22} w-(o-alkylphenyl) alkanoic acids, which originally contain high abundances of $C_{20:5}$, $C_{22:5}$ and $C_{22:6}$ fatty acids[1]. Other biomarkers that can be used to aid identification of aquatic lipids include C_{16} and C_{20} dihydroxy fatty acids[1], isoprenoid fatty acids (4,8,12-trimethyltetradecanoic acid, 2,6,10,14-tetramethylpentadecanoic [pristanic] acid and 3,7,11,15-tetramethylhexadecanoic [phytanic] acid) [1,5], and $C_{17:1}$ and $C_{19:1}$ monounsaturated fatty acids[1]. The latter are found in a variety of aquatic animals, including fish (e.g., carp and catfish), limpets, shrimps, cuttlefish, crabs and sponges[1]. These compounds are also derived from other sources, such as plants (for the dihydroxy and unsaturated fatty acids) and milk fats (for $C_{17:1}$ monounsaturated fatty acid and isoprenoid fatty acids) [1], so their co-occurrence would strengthen any identification as aquatic in origin. For artefacts that have not been heated in the past, the identification of aquatic products is still problematic[1]. The presence of isoprenoid fatty acids, which are common components of marine animals[5], and $C_{17:1}$ and $C_{19:1}$ monounsaturated fatty acids appear to be the most appropriate suite of biomarkers to use in the absence of heating[1].

1. Baeten, J., Jervis, B., De Vos, D. & Waelkens, M. Molecular evidence for the mixing of meat, fish and vegetables in Anglo-Saxon coarseware from Hamwic, UK. *Archaeometry*. **55,** 1150–1174 (2013).
2. Copley, M.S., Hansel, F.A., Sadr, K. & Evershed, R.P. Organic residue evidence for the processing of marine animal products in pottery vessels from the pre-colonial archaeological site of Kasteelberg D east, South Africa. *S. Afr. J. Sci.* **100,** 279–283 (2004).
3. Evershed, R.P., Copley, M.S., Dickson, L. & Hansel, F.A. Experimental evidence for the processing of marine animal products and other commodities containing polyunsaturated fatty acids in pottery vessels. *Archaeometry.* **50,** 101–113 (2008).
4. Hansel, F.A. & Evershed, R.P. Formation of dihydroxy acids from Z-monounsaturated alkenoic acids and their use as biomarkers for the processing of marine commodities in archaeological pottery vessels. *Tetrahedron Lett.* **50,** 5562–5564 (2009).
5. Hansel, F.A., Copley, M.S., Madureira, L.A.S. & Evershed, R.P. Thermally produced ω-(o-alkylphenyl)alkanoic acids provide evidence for the processing of marine products in archaeological pottery vessels. *Tetrahedron Lett.* **45,** 2999–3002 (2004).
